# Designed Ankyrin Repeat Protein (DARPin) Neutralizers of TcdB from Clostridium difficile Ribotype 027

**DOI:** 10.1128/mSphere.00596-19

**Published:** 2019-10-02

**Authors:** Zeyu Peng, Rudo Simeon, Samuel B. Mitchell, Junjie Zhang, Hanping Feng, Zhilei Chen

**Affiliations:** a Department of Microbial Pathogenesis and Immunology, Texas A&M University Health Science Center, College Station, Texas, USA; b Department of Biochemistry and Biophysics, Texas A&M University, College Station, Texas, USA; c Department of Microbial Pathogenesis, University of Maryland Dental School, Baltimore, Maryland, USA; U.S. Centers for Disease Control and Prevention

**Keywords:** toxin, therapeutic, infection, protein, antibody, hypervirulent, enterotoxins

## Abstract

Clostridium difficile infection (CDI) is a leading cause of hospital-acquired diarrhea. In recent decades, the emergence of the “hypervirulent” BI/NAP1/027 strains of C. difficile significantly increased the morbidity and mortality of CDI. The pathogenesis of CDI is primarily mediated by the action of two toxins, TcdA and TcdB, with TcdB being the major virulent factor in humans. In this report, we describe the engineering of a panel of designed ankyrin repeat proteins (DARPins) that potently neutralize TcdB from the BI/NAP1/027 strains (e.g., TcdB_UK1_). The most effective DARPin, D16, inhibits TcdB_UK1_ with a 50% effective concentration (EC_50_) of 0.5 nM, which is >66-fold lower than that of the FDA-approved anti-TcdB antibody bezlotoxumab (EC_50_, ∼33 nM). Competitive enzyme-linked immunosorbent assays (ELISAs) showed that D16 blocks interactions between TcdB and its receptor, chondroitin sulfate proteoglycan 4 (CSPG4). The dimeric DARPin U3D16, which pairs D16 with DARPin U3, a disrupter of the interaction of TcdB with Frizzled 1/2/7 receptor, exhibits 10-fold-to-20-fold-enhanced neutralization potency against TcdB from C. difficile strains VPI 10463 (laboratory strain) and M68 (CF/NAP9/017) but identical activity against TcdB_UK1_ relative to D16. Subsequent ELISAs revealed that TcdB_UK1_ did not significantly interact with Frizzled 1/2/7. Computation modeling revealed 4 key differences at the Frizzled 1/2/7 binding interface which are likely responsible for the significantly reduced binding affinity.

**IMPORTANCE** We report the engineering and characterization of designed ankyrin proteins as potent neutralizers of TcdB toxin secreted by a hypervirulent ribotype 027 strain of Clostridium difficile. We further show that although TcdB toxins from both ribotype 027 and VPI 10461 interact efficiently with TcdB receptors CSPG4 and Pvrl3, TcdB_027_ lacks significant ability to bind the only known physiologically relevant TcdB receptor, Frizzled 1/2/7.

## Abstract (precis)

We report the engineering and characterization of designed ankyrin proteins as potent neutralizers of TcdB toxin secreted by a hypervirulent ribotype 027 strain of Clostridium difficile. We further show that although TcdB toxins from both ribotype 027 and VPI 10461 interact efficiently with TcdB receptors CSPG4 and Pvrl3, TcdB_027_ lacks significant ability to bind the only known physiologically relevant TcdB receptor, Frizzled 1/2/7.

## INTRODUCTION

Clostridium difficile is a bacterial pathogen that causes a variety of intestinal diseases collectively referred to as C. difficile infection (CDI). The symptoms of CDI range from mild diarrhea to life-threatening pseudomembranous colitis and toxic megacolon (1–3). Each year, C. difficile causes half a million infections and ∼15,000 deaths and results in over $1 billion in treatment-associated costs in the United States, leading the CDC to declare C. difficile an urgent threat to public health (4). A major culprit responsible for CDI is believed to be the administration of broad-spectrum antibiotics, which disrupts the natural gut microflora that would otherwise suppress C. difficile proliferation (5). It is estimated that ∼7% of hospitalized patients succumb to CDI, making C. difficile among the most dangerous nosocomial pathogens (5).

Prior to 2000, CDI could be effectively controlled by treatment with additional antibiotics such as metronidazole and vancomycin (6, 7). However, disturbing trends of increased morbidity and mortality due to relapse of C. difficile-infected patients after antibiotic treatment have since emerged (8–12). These trends correlated with the emergence of the “hypervirulent” BI/NAP1/027 strains of C. difficile (11, 13, 14) (C. difficile 027), which at one point were responsible for ∼1/3 of the CDI in the United States (15). Infection with C. difficile 027 is associated with more-severe disease and a higher death rate (15). The exact reason for the increased virulence of C. difficile 027 remains enigmatic, although many factors such as antibiotic resistance, sporulation ability, and toxin production have been proposed to contribute to its virulence (12, 16–19).

The pathology of CDI is primarily due to the action of two bacterial secreted exotoxins, toxin A (TcdA) and toxin B (TcdB) (20), that target small GTPases within the host cells, leading to disruption of tight junctions, loss of colonic epithelial barrier function, and bloody diarrhea (21). Administration of spores from nontoxigenic C. difficile strain M3 was found to significantly reduce CDI recurrence (22), highlighting a pivotal role of the toxins in CDI pathology. Vaccines against C. difficile toxins are currently being actively pursued and have enjoyed some preliminary successes (23–27). However, since CDI most often afflicts elderly hospitalized patients, the efficacy of vaccine in this unique population may be less than ideal (28, 29). The TcdB-neutralizing monoclonal antibody (MAb) bezlotoxumab was found to reduce the CDI recurrence rate from 28% to 16% in a phase III clinical trial (30) and was approved by the FDA in 2016 (31). Curiously, despite its toxin neutralization ability, bezlotoxumab did not improve the initial cure rate of CDI in patients (31) and is not approved by the FDA as a treatment for CDI. Bezlotoxumab neutralizes TcdB by directly blocking its carbohydrate binding pocket and thus preventing its attachment to the colonic mucosal cells (32). Although bezlotoxumab exhibits potent neutralization activity against TcdB from a broad range of C. difficile strains, its potency is significantly (∼185-fold) weaker against toxin from C. difficile 027 than against that from laboratory strain VPI 10463 (33).

Recently, our laboratory successfully engineered several designed ankyrin repeat proteins (DARPins) with ultrapotent neutralization activity against TcdB using phage display coupled with functional screening (34). DARPin is a small non-antibody-binding scaffold that exhibits very high thermostability and low immunogenicity and has the potential to be produced at a low cost in microbial cells (35, 36). The best dimer DARPin from our previous study—DLD4—exhibited 50% effective concentration (EC_50_) values of 4 pM and 16 pM against TcdB from C. difficile strains VPI 10463 (TcdB_VPI_, ribotype 087) and M68 (TcdB_M68_, ribotype 017), respectively, representing ∼330-fold-higher and ∼30-fold-higher *in vitro* potency than bezlotoxumab (34). DLD4 consists of two monomeric DARPins, 1.4E and U3. Cryo-electron microscopy (Cryo-EM) structural studies combined with competitive enzyme-linked immunosorbent assays (ELISAs) revealed that 1.4E and U3 interfere with the interaction between TcdB and its receptors chondroitin sulfate proteoglycan 4 (CSPG4) (37) and Frizzled receptor 1/2/7 (FZD1/2/7) (38), respectively. Unfortunately, like bezlotoxumab, all of these DARPins showed significantly weaker activity against TcdB from C. difficile 027 (TcdB_UK1_). TcdB_UK1_ and TcdB_VPI_ share a high level of sequence homology (92% identical). However, critical sequence differences at the CSPG4 and FZD1/2/7 interacting regions render anti-TcdB_VPI_ DARPins powerless against TcdB_UK1_.

In this study, we performed phage panning and functional screening to identify a panel of new DARPins with significantly improved TcdB_UK1_ neutralization activity. The best DARPin, D16, neutralized TcdB_UK1_ with an EC_50_ of 0.5 nM, making it >66-fold more potent than bezlotoxumab (EC_50_ of ∼33 nM) *in vitro*. Importantly, D16 also potently neutralizes TcdB_VPI_ (EC_50_ of 5 nM) and TcdB_M68_ (EC_50_ of 1.6 nM). Competitive ELISAs showed that all our anti-TcdB_UK1_ DARPins block the toxin interaction with CSPG4. Since DARPin U3 from our previous study impedes TcdB interaction with FZD1/2/7 receptor, which binds an epitope distinct from that of CSPG4, we constructed multiple-dimer DARPins composed of D16 and U3 with different linker sizes and topologies with a view to creating synergistic blocking of toxin-receptor interactions. These dimers were expected to exhibit enhanced neutralization activity through the avidity effect. All the dimer DARPins exhibited (10-fold-to-20-fold) enhanced neutralization potency against TcdB_VPI_ and TcdB_M68_, pointing to their potential as new antitoxin biologics for treating CDI and/or preventing its recurrence.

Intriguingly, none of the constructed dimer DARPins showed enhanced neutralization activity against TcdB_UK1_. Subsequent ELISAs revealed that, unlike TcdB_VPI_, which binds strongly to both purified ectodomains of CSPG4 and FZD2, TcdB_UK1_ lacks significant ability to interact with FZD2 despite strong ability to associate with CSPG4. Consistent with this result, TcdB_UK1_ was found to be minimally toxic to Caco-2 colon epithelium cells, which express multiple Frizzled proteins, including FZD2 and FZD7 (38, 39), but lack CSPG4 (38, 40). A closer analysis of the crystal structure of TcdB Frizzled binding domain (FBD) (TcdB-FBD; PDB code: 6C0B) revealed multiple differences between TcdB_UK1_ and TcdB_VPI_ at the FBD binding interface which likely abolish or significantly weaken the TcdB_UK1_-FZD1/2/7 interaction.

## RESULTS

### Selection of TcdB-neutralizing monomer DARPins.

**FIG 1:**
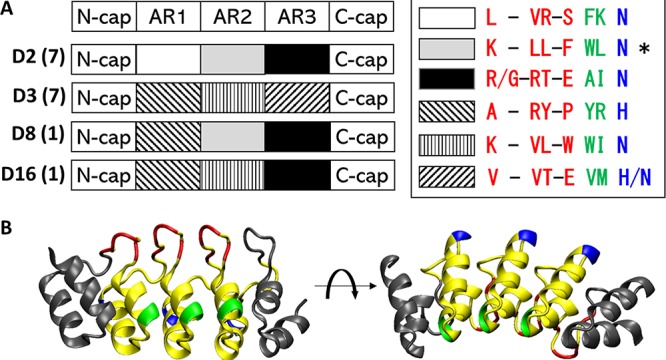
(A) Schematics of the different DARPins. The identity of the randomized residues in each repeat module is indicated in the legend on the right. The different colors represent the different positions on the ankyrin repeat module and are pictorially represented in panel B. The number of clones with the same AR configuration (with or without additional framework mutations) is shown in parentheses. The asterisk (*) indicates a DARPin that contains one framework mutation. (B) A crystal structure illustrating the structure of DARPin. Residues colored in red, green, and blue are randomized in the library.

DARPins were designed based on repeat modules of natural ankyrin proteins and consist of an N-terminal capping repeat (N-cap), three (N3C) internal ankyrin repeats (ARs), and a C-terminal capping repeat (C-cap) (41). In a DARPin library, each internal repeat contains six randomized positions on the flexible surface-exposed loop and one partially randomized position on the hinge region, yielding a total of 18 plus 3 randomized positions in each N3C DARPin. Additional mutations at the framework positions can emerge during repeated PCR amplification. A previously constructed DARPin library comprising a complex of ∼2 × 10^9^ variants was used in phage panning against biotinylated TcdB_UK1_. DARPin variants from the 4th round of panning, which showed significantly elevated levels of TcdB_UK1_ binding ability (see Fig. S1 in the supplemental material), were cloned into the pET28a vector and expressed in BL21(DE3) Escherichia coli cells and underwent functional screening. Among the 760 individual clones screened, 57 clones rescued Vero cell viability from TcdB_UK1_ toxicity by >70%. All these clones were sequenced, and 16 unique clones were identified (see Table S1 in the supplemental material). Interestingly, these 16 unique DARPins were found to be composed of only 6 distinct ARs in 4 different configurations (Fig. 1), suggesting that these DARPins likely bind to overlapping epitopes on TcdB_UK1_. Four DARPins (one from each unique configuration [D2, D3, D8, and D16]) that lacked any additional framework mutations were selected for further characterization.

10.1128/mSphere.00596-19.1 FIG S1 Purification of DARPins. Each DARPin was expressed in 5 ml of E. coli culture and purified by Ni-NTA affinity chromatography. Purified DARPins were analyzed by SDS-PAGE. Download FIG S1, TIF file, 1.2 MB. Copyright © 2019 Peng et al. 2019 Peng et al. This content is distributed under the terms of the Creative Commons Attribution 4.0 International license.

10.1128/mSphere.00596-19.5 TABLE S1 Amino acid sequences of the unique clones of anti-TcdB_UK1_ DARPins. Download Table S1, DOCX file, 0.02 MB. Copyright © 2019 Peng et al. 2019 Peng et al. This content is distributed under the terms of the Creative Commons Attribution 4.0 International license.

**FIG 2:**
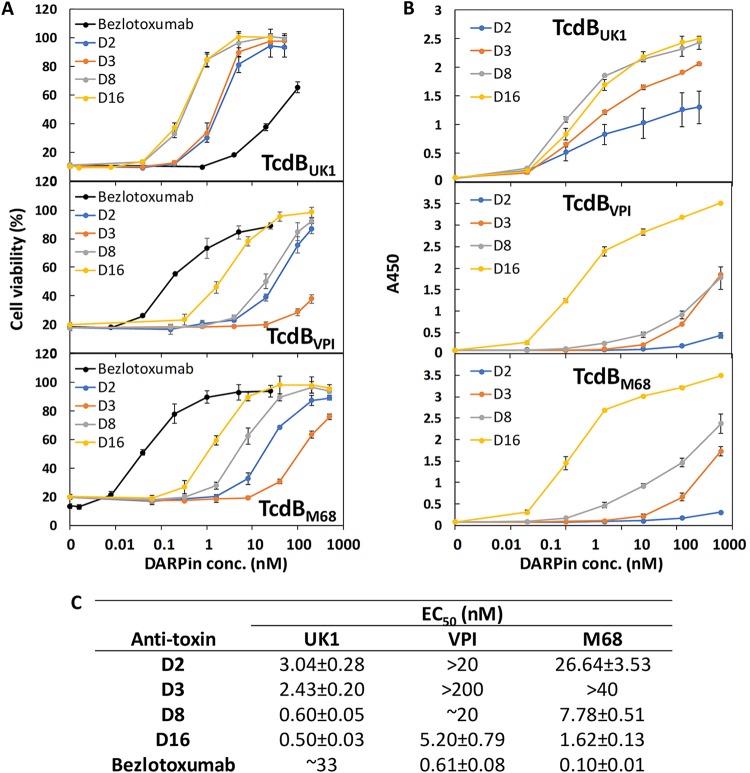
(A and B) DARPins strongly exhibited the ability to neutralize (A) and bind (B) the different TcdB toxins. conc., concentration. (C) TcdB neutralization potency of different DARPins and bezlotoxumab. For neutralization assays, serially diluted immobilized-metal affinity chromatography (IMAC)-purified DARPins were mixed with the appropriate toxin and then added to Vero cells seeded the night before in 96-well plates. The cell viability was quantified by the CellTiterGlo assay 72 h later and normalized to naive Vero cells. For ELISAs, the MaxiSorp plates were coated with the appropriate toxin followed by treatment with serially diluted DARPins. The amounts of plate-bound DARPins (containing Myc tags) were quantified using an anti-c-Myc antibody. Data in panel A represent averages of results from at least 2 independent experiments. Data presented in panel B are representative of results from two independent experiments performed in duplicate.

In addition to neutralizing TcdB_UK1_, all four DARPins also inhibited TcdB_VPI_ and TcdB_M68_ (Fig. 2A). The best DARPin, D16, exhibited an EC_50_ of 0.5 nM against TcdB_UK1_, representing a >66-fold-higher *in vitro* potency than bezlotoxumab against the same toxin. D16 also exhibited low nanomolar neutralization potency against TcdB_VPI_ (ribotype 087, EC_50_ of 5.2 nM) and TcdB_M68_ (ribotype 017, EC_50_ of 1.6 nM), representing lower potencies for these toxins relative to bezlotoxumab. The ability of the DARPins to bind the different toxins generally matches their toxin neutralization potency (Fig. 2B), with D16 being both the strongest binder and the most potent neutralizer of all the toxins.

### Mechanism of TcdB_UK1_ neutralization by monomeric DARPins.

There are three known TcdB receptors: chondroitin sulfate proteoglycan 4 (CSPG4), poliovirus receptor-like 3 (PVRL3 or NECTIN3), and members of the Frizzled protein family FZD1/2/7, which share identical sequences in the TcdB-binding region (37, 38, 42). In our previous study performed with TcdB_VPI_, most of the identified antitoxin DARPins interfered with the TcdB-CSPG4 interaction based on Cryo-EM structural studies and competitive ELISA results (34). The high frequency of antitoxin DARPin hits that block CSPG4 binding seen in our previous study likely stemmed from the use of Vero cells, which express a high level of CSPG4 (40), in our functional screening.

**FIG 3:**
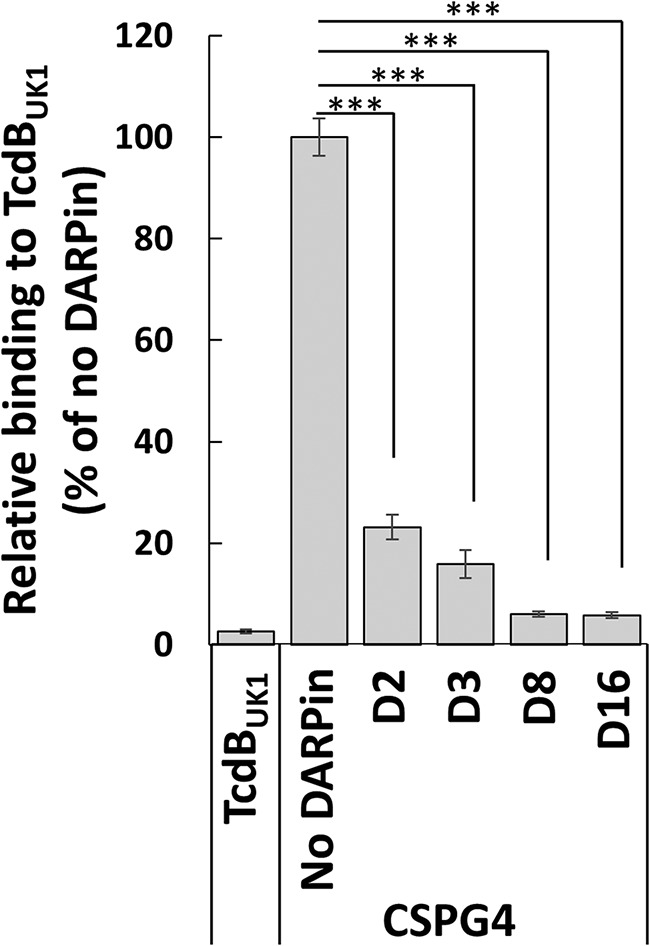
Anti-TcdB_UK1_ DARPins block the interaction between TcdB and its receptor CSPG4. ***, *P < *0.001 (*t* test). The wells of an ELISA plate were coated with TcdB_UK1_ followed by treatment with a 1 nM concentration of a CSPG4 extracellular domain-GFP fusion protein alone or in a mixture with a 250 nM concentration of the indicated DARPins. The amounts of plate-bound CSPG4 were detected using an anti-GFP antibody. The data are representative of results from two independent experiments and of averages of results from quadruplicate samples.

We repeated the competitive ELISA for the four unique DARPins (i.e., D2, D3, D8, and D16). The wells of the ELISA plate were first coated with TcdB_UK1_ (4 μg/ml) overnight at 4°C. The next day, a green fluorescent protein (GFP)-tagged extracellular domain of CSPG4 (CSPG4-EC-GFP) was added in the presence or absence of the different DARPins. The plate was incubated at room temperature for 2 h, and the amounts of bound CSPG4-EC-GFP were detected using anti-GFP antibody. As shown in Fig. 3, all four DARPins significantly reduced the binding signal from CSPG4-EC-GFP, with D16 producing the most signal reduction, indicating that all these monomer DARPins interfere with the TcdB_UK1_-CSPG4 interaction. Thus, consistent with our previous finding, inhibition of CSPG4 interaction emerged as a dominant mechanism used by antitoxin DARPins in Vero cell-based functional screening.

### Dimeric DARPins with enhanced potency against TcdB_VPI_ and TcdB_M68_.

Fusion of multiple binders to nonoverlapping epitopes has been reported to significantly enhance the overall target-binding affinity via the avidity effect (43, 44). Previously, we identified DARPin U3, which interferes with the interaction between TcdB and its receptor FZD1/2/7 (34). Dimer DARPin—DLD4—consisting of U3 and 1.4E joined by a 3× GGGGS linker exhibited >100-fold-higher neutralization potency against TcdB_VPI_ than either constituent monomer. Since both D16 and 1.4E interfere with the TcdB-CSPG4 interaction, we reasoned that a dimeric DARPin (Fig. S2B) comprising U3 and D16 joined by the same linker should exhibit stronger toxin neutralization potency than D16 alone.

10.1128/mSphere.00596-19.2 FIG S2 Schematic of monomeric and dimeric DARPins. (A) Monomeric DARPins contain an N-terminal 6×His tag and a Myc tag. The sequence from D16 is presented as an example. (B) The constituent DARPins in a dimeric DARPin are separated by a (GGGGS) × 3 linker sequence. The sequence from D16U3 is presented as an example. Download FIG S2, TIF file, 1.1 MB. Copyright © 2019 Peng et al. 2019 Peng et al. This content is distributed under the terms of the Creative Commons Attribution 4.0 International license.

**FIG 4:**
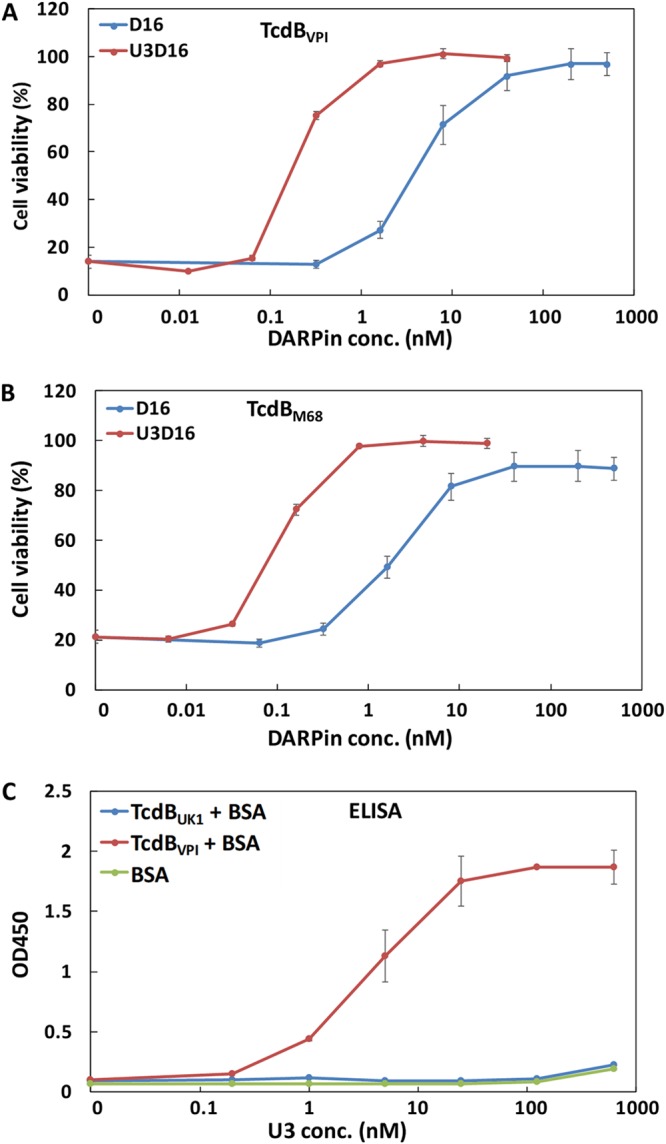
DARPin dimer U3D16 showed enhanced neutralization ability against TcdB_VPI_ (A) and TcdB_M68_ (B). Serially diluted DARPins were mixed with the appropriate toxins and then added to Vero cells that had been seeded the night before. Cell viability was quantified by the CellTiterGlo assay 72 h later and normalized to naive Vero cells. The error bars represent mean deviations of results from two independent experiments. (C) U3 lacks the ability to bind to TcdB_UK1_ as determined by ELISA. The ELISA plates were coated with the appropriate toxin and then blocked with BSA prior to the addition of serially diluted DARPin U3. The amounts of plate-bound DARPin were quantified using an anti-c-Myc antibody. The data are representative of results from two independent experiments performed in duplicate. OD_450_, optical density at 450 nm.

**FIG 5:**
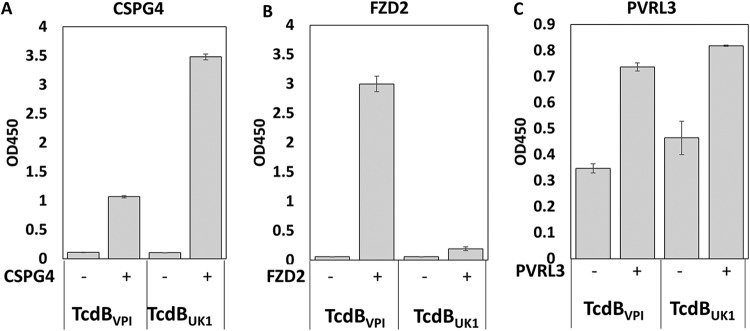
TcdB_UK1_ lacks significant ability to interact with FZD2. The wells of an ELISA plate were coated with TcdB_VPI_ or TcdB_UK1_ followed by treatment with CSPG4-EC-GFP (1 nM) (A), FZD2-Fc (4 nM) (B), or PVRL3 (100 nM) (C). The amount of plate-bound CSPG4, FZD2, or PVRL3 was detected using each of the respective antibodies. Results are representative of at least two independent experiments. Error bars represent the mean deviations of results from duplicate samples.

Indeed, dimeric DARPin U3D16 showed 10-fold-to-20-fold-higher activity toward TcdB_VPI_ and TcdB_M68_ than D16 alone (Fig. 4A and B). However, surprisingly, the TcdB_UK1_ neutralization ability shown by U3D16 was weaker than that seen with D16 alone (Fig. S3A). We subsequently prepared additional dimeric DARPins with a reverse configuration (i.e., D16U3) and/or different linker lengths (4×, 5×, and 6× GGGGS), but to no avail (Fig. 3 and 5). Furthermore, a mixture containing both D16 and U3 showed activity identical to that seen with D16 alone (Fig. S3C). These results suggest that, although U3 efficiently neutralizes TcdB_VPI_ and TcdB_M68_, it is powerless against TcdB_UK1_. Subsequent ELISAs confirmed that U3 lacks the ability to bind TcdB_UK1_ (Fig. 4C).

10.1128/mSphere.00596-19.3 FIG S3 Neutralization potency of engineered dimeric DARPins against UK1 TcdB. Purified DARPins were added to Vero cells together with TcdB_UK1_ (1 pg/ml). Cell viability was quantified by the CellTiter Glo assay 72 h later and normalized to naïve Vero cells. Experiments were conducted in triplicate. Error bars represent the standard deviation. (A) D16 versus U3D16. (B) D16 versus D16U3. (C) D16 versus mixture of D16 and U3. (D) D16 versus U3D16 and D16U3 with longer linkers. Download FIG S3, TIF file, 0.8 MB. Copyright © 2019 Peng et al. 2019 Peng et al. This content is distributed under the terms of the Creative Commons Attribution 4.0 International license.

### TcdB_UK1_ lacks significant ability to interact with FZD2.

The inability of U3 to bind/neutralize TcdB_UK1_ was surprising. DARPin U3 neutralizes TcdB by interfering with the interaction between TcdB and the FZD1/2/7 receptor (34). The members of the Frizzled family of receptors are important for Wnt signaling, a key signaling pathway that regulates cell proliferation and self-renewal (45). Unlike CSPG4, which is not present in the colon epithelium but abundant in the subepithelium layer, FZD2 and FZD7 are highly expressed in mouse and human colonic epithelium, making them the most physiologically relevant receptors for TcdB (38).

The lack of binding of U3 to TcdB_UK1_ prompted us to examine the interaction between TcdB_UK1_ and FZD2. ELISA plates were coated with TcdB_VPI_ or TcdB_UK1_ prior to the addition of the extracellular domain of CSPG4 or FZD2. After thorough washing, the amounts of plate-bound CSPG4 and FZD2 were detected using the respective antibodies. As shown in Fig. 5, CSPG4 can bind efficiently to both TcdB_VPI_ and TcdB_UK1_, with the TcdB_UK1_ interaction being the stronger. In contrast, only TcdB_VPI_ was able to significantly associate with FZD2 at the test concentration, indicating that the binding affinity of TcdB_UK1_ to FZD2 is much weaker than that shown by TcdB_VPI_. The extracellular domain of PVRL3 appeared to bind TcdB_VPI_ and TcdB_UK1_ with similar levels of affinity (Fig. 5C).

**FIG 6:**
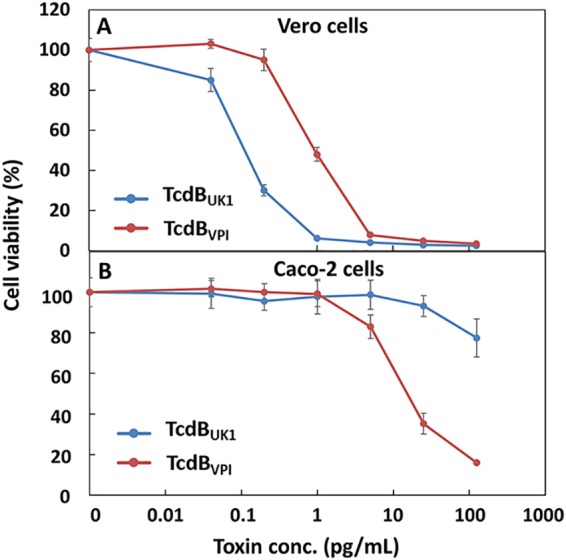
Vero and Caco-2 cells exhibit different levels of sensitivity to TcdB_UK1_ and TcdB_VPI_. Vero cells (A) or Caco-2 cells (B) were incubated with serial dilutions of TcdB_UK1_ or TcdB_VPI_. The cell viability was quantified using CellTiter-Glo reagent 72 h later and normalized to naive cells. The error bars represent standard deviations of results from two independent experiments performed in triplicate.

To interrogate the inability of TcdB_UK1_ to interact with FZD1/2/7 from a different angle, we compared the levels of toxicity of TcdB_UK1_ and TcdB_VPI_ in Caco-2 and Vero cells (Fig. 6). Caco-2 cells lack CSPG4 but express multiple Frizzled proteins, including FZD2 and FZD7 (38, 39). On the other hand, Vero cells are abundant with respect to CSPG4, FZD2, and PVRL3 (40). In Vero cells, TcdB_UK1_ (50% lethal concentration [LC_50_], 0.15 ± 0.01 pg/ml) appeared to be 6.5-fold more toxic than TcdB_VPI_ (LC_50_, 0.98 ± 0.04 pg/ml). However, in Caco-2 cells, the order was reversed and TcdB_UK1_ (LC_50_, >125 pg/ml) appeared to be far less toxic than TcdB_VPI_ (LC_50_, ∼10 pg/ml). These data indicate that TcdB_UK1_ lacks significant ability to enter cells through the FZD1/2/7 receptor and are consistent with our finding that TcdB_UK1_ exhibits poor binding affinity for FZD2 (Fig. 5).

## DISCUSSION

C. difficile infection (CDI) is the most common cause of antibiotic-associated diarrhea and gastroenteritis-associated death in developed countries. The prevalence, mortality, and costs associated with CDI make C. difficile a major threat to public health. The pathology of CDI stems primarily from the two exotoxins secreted by C. difficile bacteria, TcdA and TcdB, of which TcdB is considered the primarily virulence factor in human (46). C. difficile was first reported to cause human disease in 1978 (47, 48). In the past, CDI has been routinely treated with supportive therapy and regimens of antibiotics. However, the cure rate has been steadily decreasing over the last decades largely due to the emergence of a hypervirulent (NAP1/BI/027) strain of C. difficile (9–11).

Previously, using phage display coupled with functional screening, we successfully isolated a panel of DARPins with potent neutralization activity against TcdB from the laboratory strain of C. difficile VPI 10463 (ribotype 087) and the clinical strain M68 (NAP9/CF/017). The goal of this study was to identify strong binders/neutralizers of TcdB from hypervirulent but intractable C. difficile strain UK1 (NAP1/BI/027). The sequence of TcdB_UK1_ shares 92% and 88% similarity with those of TcdB_VPI_ and TcdB_M68_, respectively. In comparison, TcdB_VPI_ shares 93.7% sequence similarity with TcdB_M68_. TcdB from a NAP1/BI/027 strain was reported to induce a greater cytopathic effect on a variety of cell types (49) and to exhibit a substantially lower lethal dose and more-extensive brain hemorrhaging in mice than that were seen with the laboratory strain (50).

To investigate these factors, we performed four rounds of panning of phage displaying a randomized library of designed ankyrin repeat proteins (DARPins) against TcdB from the UK1 strain of C. difficile. TcdB_UK1_ binders that emerged from this approach were subjected to an *in vitro* potency screen carried out on Vero cells, resulting in the identification of a panel of DARPins with potent neutralization activity against TcdB_UK1_. DARPins are synthetic ankyrin repeat proteins composed of three internal ankyrin repeat (AR) domains sandwiched between N-capping and C-capping domains. Interestingly, the top 57 anti-TcdB_UK1_ DARPins that emerged from this study share the same 6 ARs in 4 different configurations, suggesting that these DARPins likely target regions surrounding a common epitope (Fig. 1). The four DARPins representing each unique repeat configuration and without any framework mutations were further characterized. The most effective DARPin, D16, neutralized TcdB from C. difficile strains UK1, VPI 10463, and M68 with EC_50_ values of 0.5 nM, 5.2 nM, and 1.6 nM, respectively. The *in vitro* potency of D16 toward TcdB_UK1_ is >66-fold higher than that of the toxin-neutralizing therapeutic antibody bezlotoxumab (EC_50_ of >33 nM) (Fig. 2).

All four unique anti-TcdB_UK1_ DARPins from our screen were found to block the interaction of TcdB with the receptor CSPG4 (Fig. 3), much like the antitoxin DARPins. This finding is consistent with results from our previous study in which the vast majority of the isolated anti-TcdB_VPI_ DARPins neutralized the toxin by interfering with the TcdB-CSPG4 interaction (34). There are currently three known receptors for TcdB: CSPG4, Frizzled 1/2/7, and PVRL3. Although Vero cells express high levels of all these receptors (40), the recurrent emergence of CSPG4-interfering DARPins from functional screens using Vero cells points to a dominant role of CSPG4 in mediating TcdB entry in these cells.

Previously, we identified DARPin 1.4E, which inhibited the CSPG4-TcdB_VPI_ interaction but showed neither activity toward nor binding to TcdB_UK1_ (34). Since both TcdB_VPI_ and TcdB_UK1_ bind CSPG4 (Fig. 5A), the ability of the antitoxin DARPins reported in this study to neutralize both TcdB_UK1_ and TcdB_VPI_, albeit with different potencies, indicates that these DARPins bind epitopes on TcdB that are distinct from those bound by DARPin 1.4E and that these epitopes partially overlap the footprint of CSPG4, which in part overlaps the epitope of DARPin 1.4E. Unfortunately, the data representing the binding interface of CSPG4 and DARPin 1.4E lack sufficient resolution to support detailed mutagenesis studies to elucidate the exact epitopes for these DARPins (34).

With a view to creating higher-potency antitoxin molecules, the dimeric DARPin U3D16 was created by fusing monomeric D16 to DARPin U3, which was earlier found to disrupt the interaction of TcdB from C. difficile VPI with the Frizzled 1/2/7 receptor. U3D16 exhibits 10-fold-to-20-fold-enhanced neutralization potency against TcdB_VPI_ and TcdB_M68_ relative to the D16 monomer, likely through an avidity effect (Fig. 4). However, unexpectedly, all the tested dimeric DARPins composed of U3 and D6 (which included variations in configuration and linker length) not only did not show enhanced activity but showed ∼10-fold-reduced activity (see Fig. S3 in the supplemental material). U3 targets an adjacent epitope on TcdB and blocks its interaction with FZD1/2/7 (34). Further studies showed that neither U3 nor FZD2 bound TcdB_UK1_ efficiently (Fig. 4C and 5). To corroborate this finding, we compared the levels of toxicity of TcdB_UK1_ and TcdB_VPI_ in Caco-2 cells and Vero cells. The Caco-2 cells are derived from the colon epithelium and lack detectable CSPG4 expression (38). On the other hand, Vero cells, derived from the kidney epithelium cells of an African green monkey, express all three known TcdB receptors (CSPG4, FZD2/7, and PVRL3) (40). At the same molar concentration, TcdB_UK1_ is more toxic to Vero cells than TcdB_VPI_ but is far less toxic to Caco-2 cells than TcdB_VPI_ (Fig. 6), indicating that TcdB_UK1_ lacks significant ability to enter cells via the FZD1/2/7 receptor. Our ELISA results indicated that, while TcdB_UK1_ and TcdB_VPI_ bound PVRL3 with similar affinities, TcdB_UK1_ associated more strongly with CSPG4 than TcdB_VPI_ and lacked significant ability to bind to FZD1/2/7 (Fig. 5). The higher affinity of TcdB_UK1_ than TcdB_VPI_ for CSPG4 is likely responsible for its greater toxicity in Vero cells, whereas the weaker affinity of TcdB_UK1_ for FZD1/2/7 may explain its reduced toxicity in Caco-2 cells.

**FIG 7:**
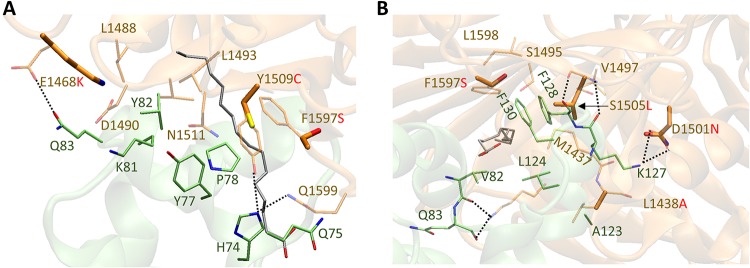
Overlay of homology model of FBD from TcdB_UK1_ with the crystal structure of TcdB_VPI_ (PDB code: 6C0B) in two different views (A, side view; B, front view). The toxin and the cysteine-rich domain 2 (CRD2) from FZD2 are represented in brown and green, respectively. Key positive allosteric modulator (PAM) (silver) binding residues from the toxin (brown) and CRD2 (green) are shown as stick models. The FBD sequence from TcdB_M68_ is identical to that from TcdB_VPI_.

Sequence alignment of TcdB_UK1_ with TcdB_VPI_ revealed six residue differences at the FZD1/2/7 binding interface (Fig. S4). Among these, we posit that four differences are most likely responsible for the weaker affinity between TcdB_UK1_ and FZD1/2/7, namely, E1468K, D1501N, Y1509C, and F1597S. The E1468 and Y1509 residues in TcdB_VPI_ form charge interactions/hydrogen bonds with Q83 and H74, respectively, in FZD2 (Fig. 7A). The presence of a positively charged Lys in position 1468 of TcdB_UK1_ instead of a negatively charged glutamic acid likely abolishes this hydrogen bond interaction. The same applies to the presence of a relatively small cysteine residue in position 1509 of TcdB_UK1_ in place of tyrosine. Residue D1501 in TcdB_UK1_ was previously shown to be critical for binding to FZD2, as the D1501A mutation abolished the ability of TcdB_VPI_ to interact with FZD2 (51). In TcdB_UK1_, position 1501 is occupied by polar residue Asn (Fig. 7B), which reduces the original two hydrogen bond interactions with K127 in FZD2 to only one. Finally, the F1597S substitution may reduce the hydrophobic interaction between Phe and the nearby F130 on FZD2. In fact, an earlier study found that the F1597G mutation in TcdB_VPI_ abolished its ability to associate with FZD2 in a pulldown assay (51). Collectively, the data indicate that there appears to be a strong structural basis for the lack of interaction between TcdB_UK1_ and FZD1/2/7.

10.1128/mSphere.00596-19.4 FIG S4 Sequence alignment of the FBD from TcdB_UK1_ and TcdB_VPI_. Key residues at the FZD binding interface are shown in blue and red. Download FIG S4, TIF file, 0.7 MB. Copyright © 2019 Peng et al. 2019 Peng et al. This content is distributed under the terms of the Creative Commons Attribution 4.0 International license.

The weakened ability of TcdB_UK1_ to use FZD1/2/7 for cell entry is surprising, as FZD7 is abundant in the colon epithelium. On the other hand, CSPG4 is absent from the colon epithelium but is predominantly expressed in multinucleated intestinal subepithelial myofibroblasts (52). Independently, the same phenomenon was recently reported by Chung et al., who noted a lack of interaction between TcdB_R20291_ (with the same amino acid sequence as TcdB_UK1_) and FZD2-Fc by Western blotting (53), and by Lopez-Urena et al., whose results demonstrated that the uptake of TcdB_VPI_ by HeLa cells (expressing both CSPG4 and Frizzled receptors [38]) was not blocked by TcdB_NPI_ (with the same protein sequence as TcdB_UK1_) (57).

The explanation for the reduced activity of the dimeric DARPin composed of U3 and D16 toward TcdB_UK1_ compared to D16 is not immediately clear. It may due to a nonspecific interaction between U3 and D16 which partially obscures the target binding interface on D16. The presence of a binding partner of U3 on TcdB_VPI_ and TcdB_M68_ draws U3 away from D16, enabling the two DARPins to simultaneously bind the toxin and enhancing the neutralization potency.

In summary, we identified a panel of DARPins with potent neutralization potency toward TcdB from the hypervirulent UK1 strain of C. difficile (NAP1/BI/027). These DARPins neutralize TcdB by blocking its interaction with the receptor CSPG4. We further showed that TcdB_UK1_ does not strongly associate with FZD1/2/7, bringing into question the significance of this receptor in CDI caused by ribotype 027 hypervirulent strains of C. difficile.

## MATERIALS AND METHODS

### Protein expression and purification.

TcdB_UK1_ was recombinantly expressed in Bacillus megaterium cells and purified via the use of a nickel-nitrilotriacetic acid (Ni-NTA) column essentially as described previously (54). The fractions containing TcdB were combined and concentrated and subjected to buffer exchange using phosphate-buffered saline (PBS) (10-fold dilution of 10× PBS [Fisher catalog no. BP3991]) and ultrafiltration units (Amicon; molecular-weight cutoff [MWCO], 100 kDa). Protein purity was confirmed using SDS-PAGE. The concentration of purified protein was determined by calculating the absorbance at 280 nm with a theoretical extinction coefficient of 293,620 M^−1 ^cm^−1^. We typically obtain ∼2 mg of purified toxin per liter of culture. The purified protein was stored at −20°C in 50% glycerol. TcdB_VPI_, TcdB_M68_, CSPG4-EC-GFP, and bezlotoxumab were recombinantly expressed and purified as described previously (34, 54).

### Phage panning and functional screening.

An in-house N3C DARPin library with a diversity level of ∼10^9^ was used in the phage panning essentially as described previously (34, 55). Purified TcdB_UK1_ was biotinylated via the use of EZ-Link-Sulfo NHS-LC [succinimidyl 6-(biotinamido)hexanoate] biotin (Pierce) and used as the target protein in four rounds of sequential phage panning. A significant level of TcdB_UK1_ binding enrichment was observed after 4 rounds of selection using phage ELISA (55), indicative of successful phage panning.

DARPin variants from the fourth-round phage library were cloned into pET28a vector (containing an N-terminal His tag and a Myc tag; see Fig. S2A in the supplemental material) via the use of BamHI and HindIII restriction sites. A total of 760 individual E. coli BL21(DE3) clones were picked and grown in v-bottom 96-well plates (200 μl/well) in Luria broth (LB) supplemented with kanamycin (50 μg/ml) at 37°C with shaking for ∼18 h. Cells were harvested by centrifugation (1,048 × *g* for 10 min at 4°C). Each cell pellet was resuspended in 200 μl lysis buffer (PBS supplemented with 1 mM CaCl_2_, 0.5 mM EDTA, and 200 μg/ml lysozyme) and incubated at 37°C for 30 min. These cells then underwent 2 cycles of freeze-thaw between −80°C and 37°C and centrifugation at 1,048 × *g* for 20 min at 4°C. The soluble cell lysates (2 μl/well) were added to Vero cells that had been seeded the previous day at 1,500 cells/well together with TcdB_UK1_ (1 pg/ml final concentration) in 200 μl complete growth medium (Dulbecco’s modified Eagle’s medium [DMEM] supplemented with 10% fetal bovine serum, 1× nonessential amino acids, and 1× antibiotic antimycotic (Life Technologies catalog no. 15240062). The plates were incubated at 37°C and 5% CO_2_ for 72 h, and the viability of these Vero cells was quantified by the use of CellTiter-Glo reagent (Promega) and normalized to that of naive Vero cells.

Candidate DARPin clones were grown in 5 ml autoinduction medium (6 g/liter Na_2_HPO_4_, 3 g/liter KH_2_PO_4_, 20 g/liter tryptone, 5 g/liter yeast extract, 5 g/liter NaCl, 0.6% [vol/vol] glycerol, 0.05% [wt/vol] glucose, 0.2% [wt/vol] lactose) supplemented with 50 μg/ml kanamycin at 37°C with shaking for ∼18 h. These DARPins were purified by the use of Ni-NTA beads and subjected to buffer exchange using PBS and Zeba desalting columns (Thermo Scientific catalog no. 89877). Typically, 5 ml of bacterial culture yields ∼100 μg of purified DARPin. Protein purity was estimated to be >90% based on SDS-PAGE results (Fig. S1).

### *In vitro* toxicity and neutralization assay.

To compare the levels of toxicity of the different toxins (i.e., TcdB_UK1_, TcdB_VPI_, and TcdB_M68_), purified toxins were serially diluted and added to Vero cells or Caco-2 cells that had been seeded in 96-well plates the day before at 1,500 cells/well. The plates were incubated at 37°C and 5% CO_2_, and the viability of these cells was quantified 72 h later using CellTiter-Glo reagent. The minimum concentrations of the toxin that led to <20% Vero cell viability were determined to be 1 pg/ml for TcdB_UK1_, 5 pg/ml for TcdB_VPI_, and 5 pg/ml for TcdB_M68_.

To determine the neutralization potencies of the different DARPins, serially diluted DARPins were added to Vero cells (seeded at 1,500 cells/well the day before) together with the minimum dose of the appropriate toxin that led to <20% cell viability.

### Enzyme-linked immunosorbent assay (ELISA).

To compare the abilities of the different DARPins to bind TcdB_UK1_, the wells of MaxiSorp immunoplates (Nunc) were coated with 100 μl of TcdB_UK1_ (4 μg/ml) at 4°C overnight. The next day, the wells were washed and blocked with PBSTB buffer (PBS supplemented with 0.1% Tween 20 and 0.2% bovine serum albumin [BSA]) and were then incubated with serially diluted DARPins for 2 h at room temperature. After thorough washing, the amount of bound DARPin was quantified using mouse anti-c-Myc antibody (Invitrogen catalog no. 13-2500) (0.5 μg/ml) and horseradish peroxidase (HRP)-conjugated goat anti-mouse antibody (Jackson Immuno Research catalog no. 115-035-146) (0.13 μg/ml) followed by color development using 3,3′,5,5′-tetramethylbenzidine (TMB).

To determine the interaction between CSPG4/FZD2/PVRL3 and TcdB_UK1/VPI_, the immunoplates were first coated with the appropriate toxin as described above. After blocking and thorough washing, CSPG4-EC-GFP (1 nM), a GFP-tagged extracellular domain of CSPG4 (34); FZD2-Fc (R&D Systems catalog no. 1307-FZ-050) (4 nM); or PVRL3 (Sino Biological catalog no. 10852-H08H) (100 nM) was added to each well alone or in the presence of the relevant DARPin (250 nM) followed by incubation at room temperature for 2 h. After thorough washing, the amount of bound CSPG4-EC-GFP was determined using rabbit anti-GFP antibody (Proteintech catalog no. 50430-2-AP) (0.08 μg/ml) plus HRP-conjugated goat anti-rabbit antibody (Santa Cruz Biotechnology catalog no. SC-2004) (0.8 μg/ml), and the bound FZD2-Fc amount was determined using HRP-conjugated goat anti-human antibody (Jackson Immuno Research catalog no. 109-035-088) (0.2 μg/ml), while the bound PVRL3 was detected using a goat anti-PVRL3 antibody (R&D Systems catalog no. AF3064) plus HRP-conjugated donkey anti-goat antibody (Santa Cruz Biotechnology catalog no. SC-2020) followed by color development using TMB.

### Modeling study.

A homology model of TcdB_UK1_ Frizzled binding domain (FBD; amino acids 1284 to 1803) was constructed using SWISS-MODEL and was overlaid onto the FBD of TcdB_VPI_ (PDB code: 6C0B). The complex was visualized using Visual Molecular Dynamics (VMD) (56).

### Data availability.

The sequences of our engineered proteins are presented in Table S1.
