# Supplementary figures and images for: Designed Ankyrin Repeat Protein (DARPin) Neutralizers of TcdB from Clostridium difficile Ribotype 027
Source: mSphere. 2019 Oct 2;4(5):e00596-19. doi: 10.1128/mSphere.00596-19 (PMC6796971; doi:10.1128/mSphere.00596-19)

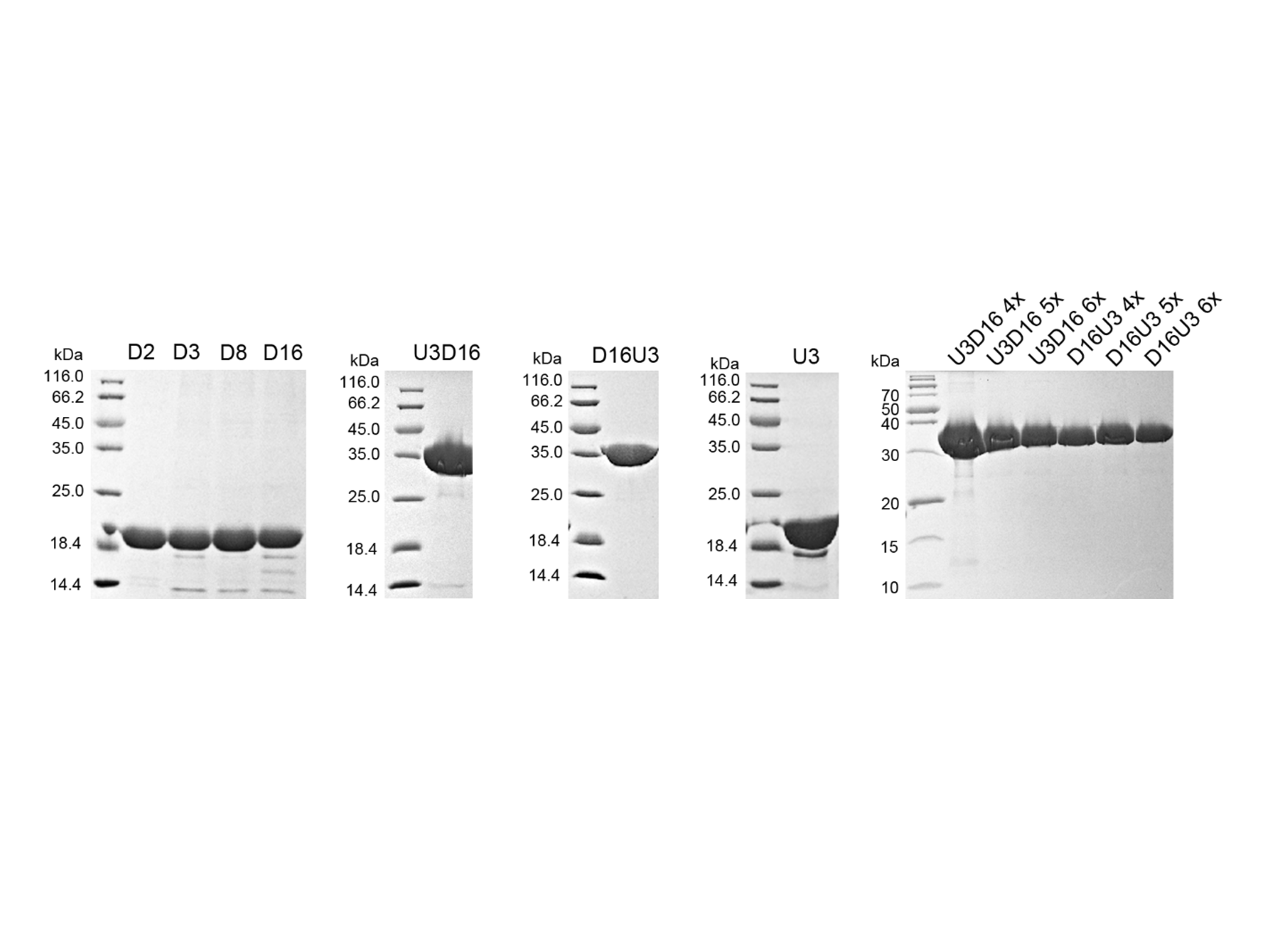

Supplement: FIG S1 [file mSphere.00596-19-sf001.tif]

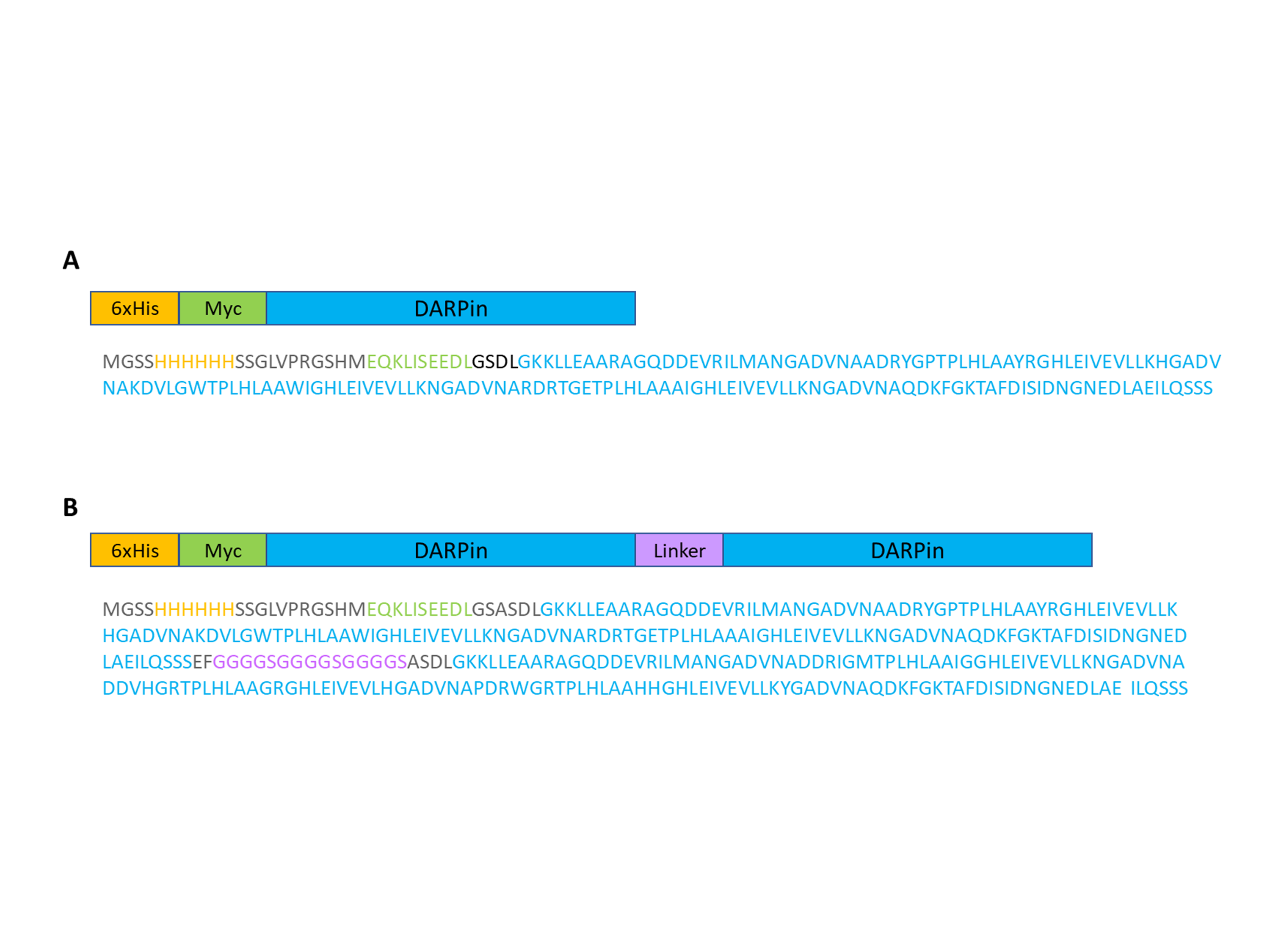

Supplement: FIG S2 [file mSphere.00596-19-sf002.tif]

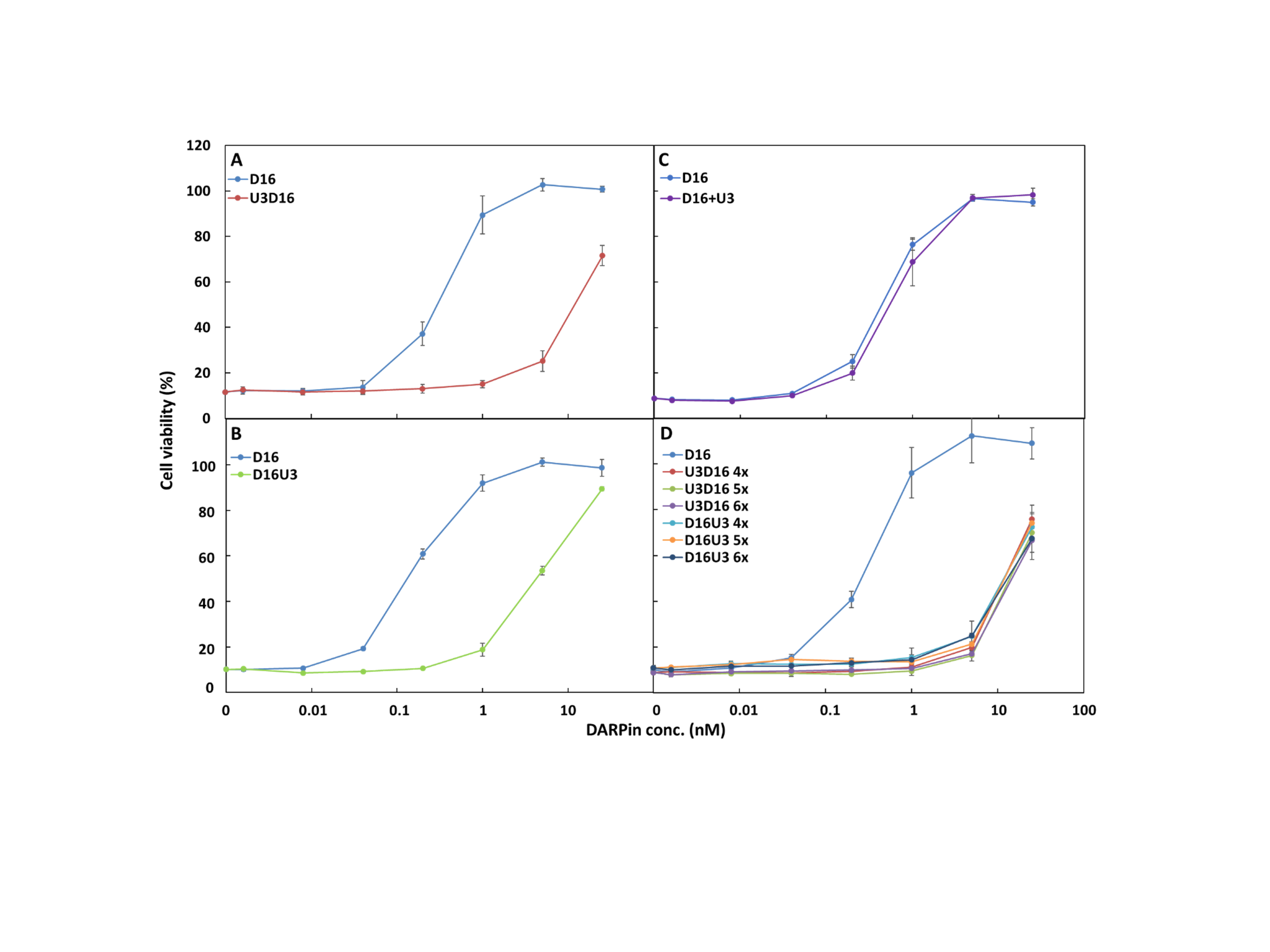

Supplement: FIG S3 [file mSphere.00596-19-sf003.tif]

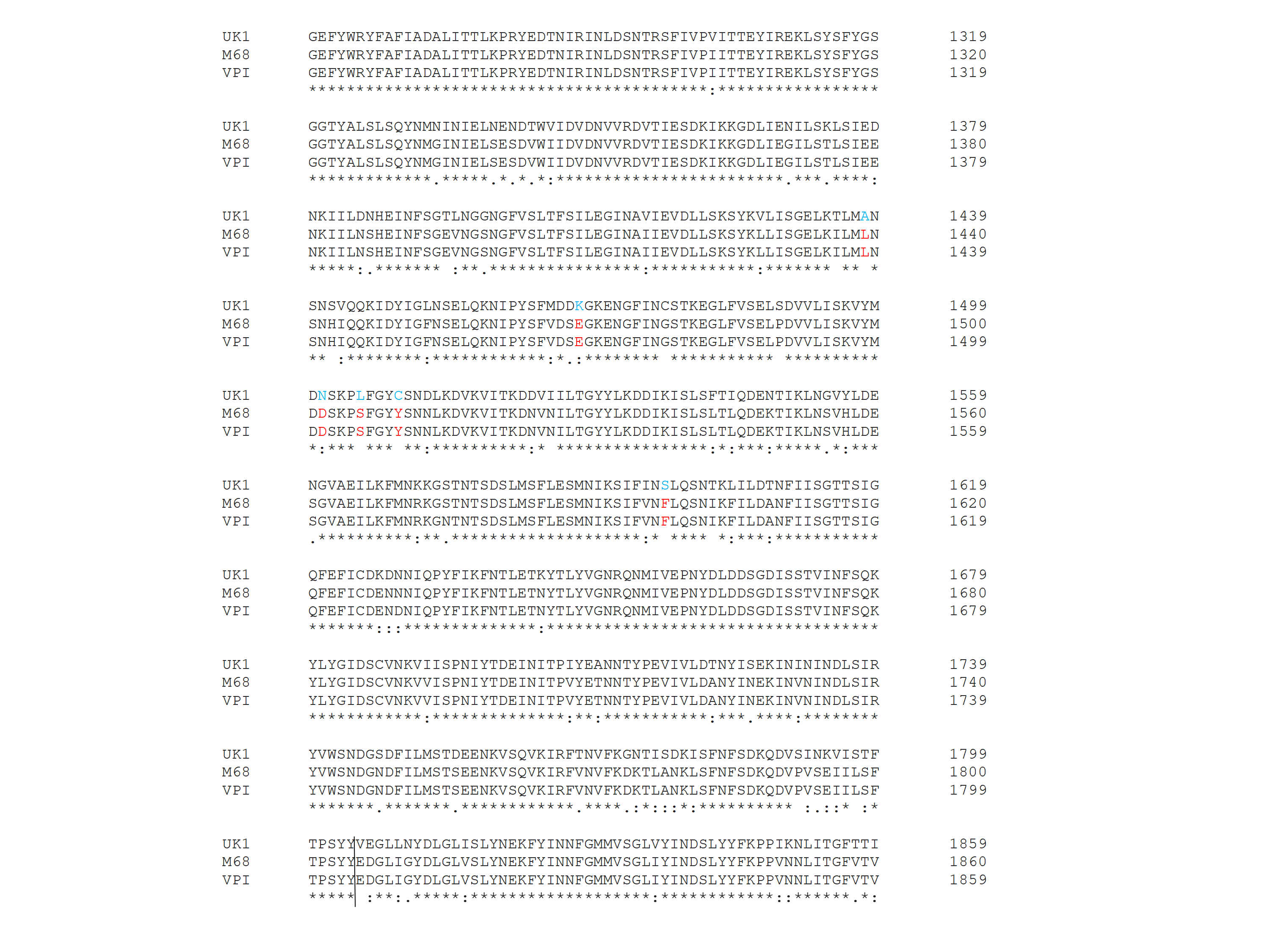

Supplement: FIG S4 [file mSphere.00596-19-sf004.tif]
