# Supplementary material for: Designed Ankyrin Repeat Protein (DARPin) Neutralizers of TcdB from Clostridium difficile Ribotype 027
Source: mSphere. 2019 Oct 2;4(5):e00596-19. doi: 10.1128/mSphere.00596-19 (PMC6796971; doi:10.1128/mSphere.00596-19)
Supplement: TABLE S1 [file mSphere.00596-19-st001.docx]

| **Motif** | **NCAP** / **AR1** / **AR2** / AR3 / **CCAP** / |
| --- | --- |
| Clone | GKKLLEAARAGQDDEVRILMANGADVNA/XDXXGXTPLHLAAXXGHLEIVEVLLKXGADVNA/XDXXGXTPLHLAAXXGHLEIVEVLLKXGADVNA/XDXXGXTPLHLAAXXGHLEIVEVLLKXGADVNA/QDKFGKTAFDISIDNGNEDLAEILQ |
| 3 | ----------------------------/A-RY-P-------YR-----------H------/K-VL-W-------WI-----------N------/V-VT-E-------VM-----------H------/------------------------- |
| 10 | ----------------------------/A-RY-P-------YR-----------H------/K-VL-W-------WI-----------N------/V-VT-E-------VM-----A-----H------/------------------------- |
| 13 | -------------------V--------/A-RY-P-------YR-----------H------/K-VL-W-------WI-----------N------/V-VT-E-------VM-----------H------/------------------------- |
| 7 | ----------------------------/A-RY-P-------YR-----------H------/K-VL-W-------WI--P--------N------/V-VT-E-------VM-----------H------/------------------------- |
| 9 | ----------------------------/A-RY-P-------YR-----A-----H------/K-VL-W-------WI---G-------N------/V-VT-E-------VM-----------H------/------------------------- |
| 6 | ----------------------------/A-RY-P-------YR-----------H------/K-VL-W-------WI---------P-N------/V-VT-E-------VM-----------H------/------------------------- |
| 15 | ----------------------------/A-RY-P-------YR-----------H------/K-VL-W-------WI-----------N------/V-VT-E-------VM-----------N------/------------------------- |
| 16 | ----------------------------/A-RY-P-------YR-----------H------/K-VL-W-------WI-----------N------/R-RT-E-------AI-----------N------/------------------------- |
| 8 | ----------------------------/A-RY-P-------YR-----------H------/K-LL-F--V----WL-----------N------/R-RT-E-------AI-----------N------/------------------------- |
| 14 | ----------------------------/L-VR-S-------FK-----------N------/K-LL-F--V----WL-----------N------/G-RT-E-------AI-----------N------/------------------------- |
| 1 | ----------------------------/L-VR-S-------FK-----------N------/K-LL-F--V----WL---------P-N------/R-RT-E-------AI-------I---Y------/------------------------- |
| 2 | ----------------------------/L-VR-S-------FK-----------N------/K-LL-F--V----WL-----------N------/R-RT-E-------AI-----------N------/------------------------- |
| 4 | ------------G---------------/L-VR-S-------FK-----------N------/K-LL-F--V----WL-----------N------/R-RT-E-------AI-----------N------/------------------------- |
| 12 | ----------------H-----------/L-VR-S-------FK-----------N------/K-LL-F--V----WL-----------N------/R-RT-E-------AI-----------N------/------------------------- |
| 5 | ----------------------------/L-VR-S-------FK-----------N----D-/K-LL-F--V----WL-----------N------/R-RT-E-------AI-----------N------/--------S---------------- |
| 11 | -----G----------------------/L-VR-S-------FK-----------N------/K-LL-F--V----WL-----------N------/R-RT-E-------AI----------RN------/------------------------- |

**Table S1. Amino acid sequences of the unique clones of anti-TcdB_UK1_ DARPins.**
